# Boron Toxicity Causes Multiple Effects on *Malus domestica* Pollen Tube Growth

**DOI:** 10.3389/fpls.2016.00208

**Published:** 2016-02-26

**Authors:** Kefeng Fang, Weiwei Zhang, Yu Xing, Qing Zhang, Liu Yang, Qingqin Cao, Ling Qin

**Affiliations:** Beijing Key Laboratory for Agricultural Application and New Technique, College of Plant Science and Technology, Beijing University of AgricultureBeijing, China

**Keywords:** *Malus domestica*, boron toxicity, calcium, actin, callose, pectin, arabinogalactan proteins

## Abstract

Boron is an important micronutrient for plants. However, boron is also toxic to cells at high concentrations, although the mechanism of this toxicity is not known. This study aimed to evaluate the effect of boron toxicity on *Malus domestica* pollen tube growth and its possible regulatory pathway. Our results showed that a high concentration of boron inhibited pollen germination and tube growth and led to the morphological abnormality of pollen tubes. Fluorescent labeling coupled with a scanning ion-selective electrode technique detected that boron toxicity could decrease [Ca^2+^]c and induce the disappearance of the [Ca^2+^]c gradient, which are critical for pollen tube polar growth. Actin filaments were therefore altered by boron toxicity. Immuno-localization and fluorescence labeling, together with fourier-transform infrared analysis, suggested that boron toxicity influenced the accumulation and distribution of callose, de-esterified pectins, esterified pectins, and arabinogalactan proteins in pollen tubes. All of the above results provide new insights into the regulatory role of boron in pollen tube development. In summary, boron likely plays a structural and regulatory role in relation to [Ca^2+^]c, actin cytoskeleton and cell wall components and thus regulates *Malus domestica* pollen germination and tube polar growth.

## Introduction

Boron is an essential micronutrient for the normal development of higher plants (Blevins and Lukaszewski, 1998). Its main role is to form borate-diol ester bonds to link two rhamnogalacturonan II (RGII) chains of pectic polysaccharide (Pérez-Castro et al., 2012; Funakawa and Miwa, 2015). Recent research illustrated that boron also cross-link glycosylinositol phosphorylcer amides of the plasma membrane with arabinogalactan proteins (AGPs) of the cell wall, thereby attaching the membrane to the cell wall (Tenhaken, 2014; Voxeur and Fry, 2014). Thus, boron is known to affect the mechanical properties of the cell wall (Dumont et al., 2014). There is a narrow range of favorable boron concentrations for plant development. Abnormal levels of boron can be toxic or can trigger deficiency symptoms (Pérez-Castro et al., 2012). The optimum boron level for one species can be either toxic or insufficient for other species (Blevins and Lukaszewski, 1998). Boron toxicity is an important agricultural problem that limits crop productivity (Nable et al., 1997) and attracts increase interest. Boron toxicity has been shown to affect several developmental or biochemical processes in plants (Sakamoto et al., 2011), including inhibiting the formation of glutathione (Ruiz et al., 2003) and tocopherol (Keles et al., 2004), reducing root cell division (Aquea et al., 2012), and shoot cell wall expansion (Loomis and Durst, 1992), decreasing fruit number, size and weight, formatting reactive oxygen species(ROSs) (Paull et al., 1992; Nable et al., 1997; Karabal et al., 2003; Cervilla et al., 2007), increasing oxidative damage (Gunes et al., 2006; Molassiotis et al., 2006; Sotiropoulos et al., 2006), affecting the photosynthesis and antioxidant apparatus (Landi et al., 2013), and leading to DNA damage (Sakamoto et al., 2011). A cDNA-AFLP analysis revealed that long-term boron stress induced changes related to signal transduction, metabolism of carbohydrate, energy, nucleic acid, protein, amino acid and lipid, cell wall and cytoskeleton modification, stress responses, and cell transport in *Citrus grandis* and *Citrus sinensis* (Guo et al., 2014). Furthermore, several genes involved in plant tolerance to boron stress have been identified, including *Arabidopsis thaliana BOR4* and *TIP5;1* (Miwa et al., 2007; Pang et al., 2010), barley (*Hordeu mvulgare*) *Bot1* (Sutton et al., 2007), wheat and barley *HvBOR2* (Reid, 2007), and citrus (*Citrus macrophylla*) *CmBOR1* (Cañon et al., 2013). These genes encode transport molecules that exclude excess boron or regulate intracellular boron homeostasis to prevent boron stress (Sakamoto et al., 2011). Although a number of studies have been performed in this field, as described above, the effects of boron toxicity on sexual plant reproduction remain largely unknown.

Pollen tubes represent a fast growing system that requires boron to germinate and maintain tube elongation (Taylor and Hepler, 1997), making it a good system with which to investigate the influence of boron toxicity. Pollen tubes are cells that grow from their tips, whose elongation exhibits a polarized pattern (Hao et al., 2013). During the process of pollen tube growth, large amounts of membrane, and cell wall precursors are transported by the secretory vesicles derived from the Golgi apparatus to the tip to form the new cell wall and thus lead to pollen tube elongation (Taylor and Hepler, 1997; Ketelaar et al., 2008; Moscatelli and Idilli, 2009; Zhang et al., 2010; Bou and Geitmann, 2011). The pollen tube wall is mainly composed by cellulose, callose, and pectins, among which the pectins seem to be the major component of the cell wall (Li et al., 1996, 2002; Ferguson et al., 1998). The Golgi apparatus produced esterified pectin residues and the latter is secreted at the extreme apex of the pollen tube (Hasegawa et al., 1998). The esterified pectins are de-esterified by the enzyme pectin methyl-esterase (PME) when arrival at the cell wall (Geitmann, 1999; Li et al., 2002). De-esterification of pectin produces acidic residue which cross-links Ca^2+^ ions to form a semi rigid pectate gel (Braccini and Perez, 2001), thus providing mechanical support for the elongating tube. Esterified pectins are mainly present at the apex and speculated to allow tube expansion (Franklin-Tong, 1999). Therefore, de-esterified and esterified pectins control jointly the growth of plant cell (Wolf and Greiner, 2012).

Boron is necessary for pollen tubes (Obermeyer et al., 1996), and affects pollen tube morphology and tube growth (Dickinson, 1978; Holdaway-Clarke and Hepler, 2003). However, few data are available on the effects of boron toxicity on pollen germination and tube growth. The detailed regulatory effects of boron toxicity on pollen tube development remain to be elucidated.

In the present study, *Malus domestica* pollen was chosen as the material with which to study the influence of boron toxicity on germination and pollen tube growth, focusing on the dynamics of calcium, actin, and cell wall components. Our results revealed that boron toxicity could interrupt the calcium gradient at the tip of a pollen tube and block its polar growth, likely via disturbing the actin organization and thus disturbing the cell wall material directional transportation and cell wall construction.

## Materials and Methods

### Plant Materials and Pollen Culture

Mature pollen grains were collected from *Malus domestica* trees grown in Henan Province on April 10, 2014. The collected pollen grains were dried on paper towels and then stored in vials at –20°C until use.

The basal medium for pollen tube growth was composed by 20% (w/v) sucrose and 0.01% CaCl_2_, pH 6.8. Pollen grains was placed into culture medium in concentration of 1.0 mg mL^–1^. Different concentrations of boric acid (Sigma, St. Louis, MO, USA) was added to the medium at the beginning of incubation. The culture with shake for 100 rpm at 30°C in the darkness.

Method of Dafni (2000) was used to determine the pollen germination rates under BX51 microscope which is equipped with a CoolSNAP HQ CCD camera (Photometrics) after 2 h of incubation. The lengths of pollen tubes were measured using MetaMorph (Universal Imaging) after 2 h of incubation. All experiments were performed in triplicate and at least 150 pollen tubes were measured in each experiment. Viability of the pollen tube was detect with fluorescein diacetate (FDA) according to Chebli et al. (2013).

### Measurement of Extracellular Ca^2+^Influx

Net Ca^2+^ fluxes of pollen tubes were measured in the Younger USANMT Service Center (Xuyue Beijing) using a Non-invasive Micro-test Technique (NMT-YG-100, Younger USALLC, Amherst, MA01002, USA) with the ASET 2.0 (Sciencewares, Falmouth, MA 02540, USA) and the iFluxes 1.0 (Younger USA, LLC, Amherst, MA 01002, USA) software packages (Wang et al., 2013). Excel sheet (Microsoft) was employed to analyze the obtained data and convert data into ion influx (pmol cm^–2^ sec^–1^) accordingly.

### Labeling of Cytoplasmic [Ca^2+^]c

Fluo-3/AM ester was loaded into pollen tubes to label cytoplasmic [Ca^2+^]c at low temperature in the dark at a final concentration of 10 μM, as described previously (Zhang and Renzel, 1998). After 1 h of incubation, the pollen tubes were washed with standard medium several times and placed under room temperature for 1 h. After that the pollen tubes photographed using a Leica TCS SP5 laser-scanning confocal microscope (LSCM) (Leica Co., Germany) with excitation at 488 nm and emission at 515 nm.

### Fluorescent Labeling of Actin Filament

Fluorescent labeling of actin filament was according to Hao et al. (2013). Control and boron toxicity treated pollen tubes were fixed in a freshly prepared solution of 4% paraformaldehyde in PBS (pH 6.9) for 1.5 h at room temperature, followed by three washes with PBS, and treated with enzyme solution containing 1% cellulase R-10 and 1% pectinase at 37°C for 15 min. Then the pollen tubes were washed in PBS, and incubated in 1% Triton X-100 at room temperature for 1 h. After three times with PBS, the pollen tubes were incubated in 0.2 μM phalloidin-FITC (Sigma, USA) in PBS (pH 6.9) buffer for 2 h in darkness (Hao et al., 2013). Then, the pollen tubes were washed with PBS and observed under the LSCM with Excitation at 488 nm and emission at 515 nm.

### Localization and Analysis of Cell Wall Components

Calcofluor was employed to stain cellulose as described by Lazzaro et al. (2003), callose was stained with 0.05% aniline blue according to Chen et al. (2007). The stained pollen tubes were observed and photographed under a FSX100 microscope (Olympus, Japan). Method described by Chen et al. (2007) was used to label pectins and AGPs of pollen tubes. The labeled pollen tubes were observed under the LSCM with excitation at 488 nm and emission at 515 nm. Values for fluorescence intensity was analyzed according to Chebli et al. (2013). At least 10 tubes were analyzed for each treatment, which was repeated three times. Fourier Transform Infrared (FTIR) spectroscopy analysis of wall components was performed according to Hao et al. (2013). At least 10 tubes were analyzed for each treatment, which was repeated three times.

## Results

### Boron Toxicity Affected Pollen Germination and Tube Growth

**FIGURE 1:**
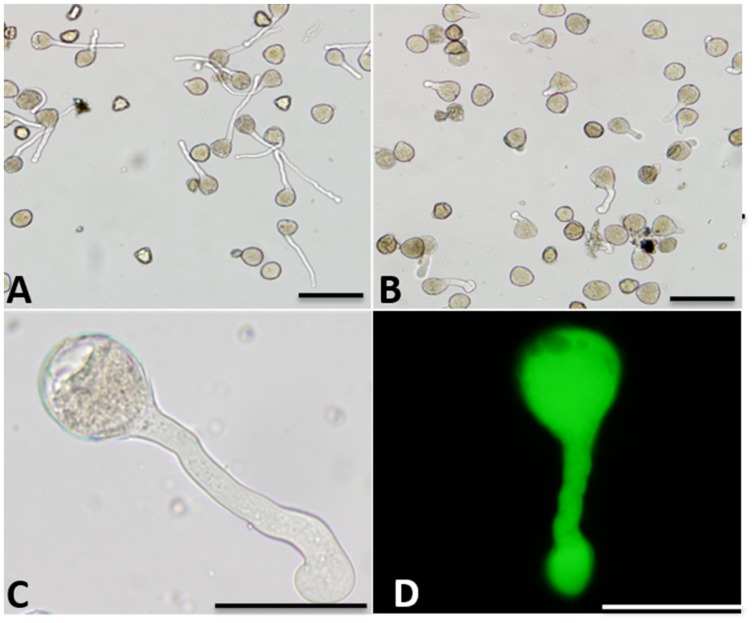
**Morphology of *Malus domestica* pollen tube under boron toxicity. (A)** Morphology of a control pollen tube with slender diameter and straight shape. **(B)** Morphology of a pollen tube treated with 0.2% boric acid, showing an abnormal tube. **(C)** Abnormal pollen tubes showing the twisted morphology and swollen tip, respectively. **(D)** Fluorescein diacetate (FDA) fluorescence indicated viability of the swollen pollen tube. Scale bar: 50 μm.

Boron affected pollen tube morphology (**Figure 1**). In germination medium including 20% sucrose, 0.015% CaCl_2_ and 0.01% H_3_BO_3_, pollen tubes appeared healthy with a regular shape. The constant diameter is illustrated in **Figure 1A**. The morphology of pollen tubes treated with high concentrations of boron was abnormal: the pollen tube was short, the tip of the tube swelled, and the diameter of the tube increased (**Figures 1B,C**). Strong FDA fluorescence indicated the viability of the swollen tube (**Figure 1D**).

**FIGURE 2:**
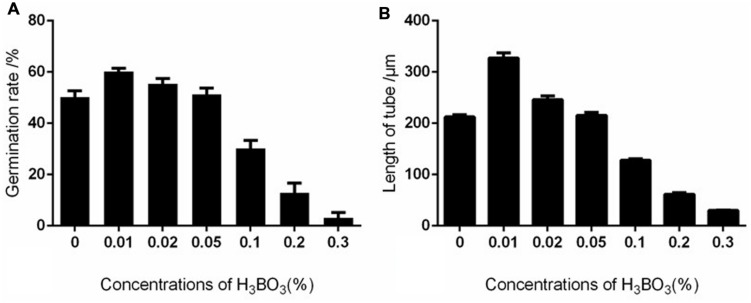
**Effect of different concentrations of boric acid on *Malus domestica* pollen germination and tube length. (A)** Effect of different concentrations of boric acid on pollen germination. **(B)** Effect of different concentrations of boric acid on tube growth.

Boron affects pollen germination and tube growth in a dose-dependent manner. Apple pollen grain has been reported to contain 55.45 μg/g boron (Gao et al., 2014), and our results showed that the endogenous levels were able to support pollen germination. At low concentrations, boron stimulated pollen germination and tube growth. Above 0.02%, boron inhibited pollen germination and tube growth. In the presence of 0.2% boric acid, the germination percentage was 12.87%, much lower than the 60.25% germination of the control pollen grains (**Figure 2A**). The average growth rate of pollen tubes treated with 0.2% boric acid was distinctly slower than for the control: the average growth rate of control pollen tubes was 163.7 μm/h, whereas the growth rate was only 30.65 μm/h in the presence of 0.2% boric acid (**Figure 2B**).

### Boron Toxicity Induced a Decrease in [Ca^2+^]c Concentration and Disappearance of the [Ca^2+^]c Gradient

**FIGURE 3:**
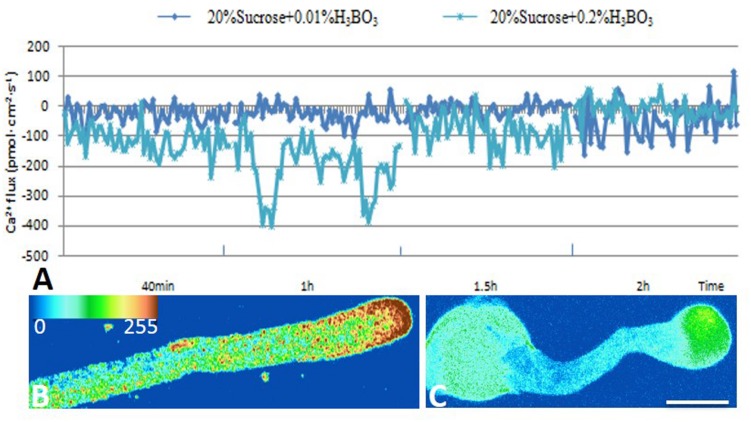
**Effect of boron toxicity on the influx of calcium at the apex of *Malus domestica* pollen tube and [Ca^2+^]c**. Bar = 25 μm. **(A)** Influx of calcium in the apex of a pollen tube at different time points. The blue line represents the CK, and the green line represents pollen treated with 0.2% boric acid. **(B)** The [Ca^2+^]c gradient at the apex of the control pollen tube. **(C)** Very weak fluorescence was detected at the apex of the pollen tube under boron toxicity, indicating the disappearance of the Ca^2+^ gradient.

Ca^2+^ influx was measured at the extreme apex of the growing pollen tubes using a vibrating electrode technique (non-invasive micro-test technique). The results showed that Ca^2+^ influx was equal to eﬄux in the control tube apex at 2 h after culture. The magnitude of Ca^2+^ influx at the extreme apex was increased upon 0.2% boric acid treatment (**Figures 3A,B**).

Furthermore, [Ca^2+^]c was detected using Fluo-3/AM in pollen tubes. The control pollen tube tips showed a representative [Ca^2+^]c gradient within 20–30 μm (**Figure 3B**), while the pollen tubes treated with 0.2% boric acid showed very weak [Ca^2+^]c fluorescence in their swollen tips compared to the control, and the [Ca^2+^]c distribution was totally altered, (**Figure 3C**, **Supplementary Figure S1a**) indicating that boron toxicity led to the disappearance of the [Ca^2+^]c gradient.

### Boron Toxicity Varied the Actin Filaments

**FIGURE 4:**
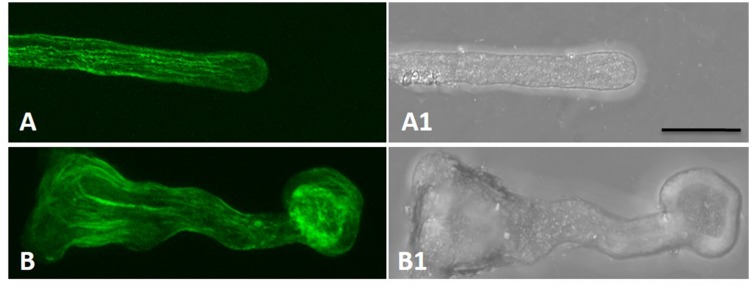
**Actin filaments of *Malus domestica* pollen tube in normal and boron toxicity media. (A)** Actin filament paralleled with the pollen tube in the normal culture medium. **(A1)** Corresponding bright field image of **(A)**. **(B)** Actin filaments showed serious fracture at the apical part of the pollen tube treated with0.2% boric acid. **(B1)** Corresponding bright field image of B. Scale bar: 25 μm.

Actin filaments take an active part in vesicle trafficking, cell wall construction, and tip growth of pollen tubes (Hao et al., 2013). Thus, the actin cytoskeleton in control and boron toxicity-treated pollen tubes was compared. As observed by LSCM, the actin filaments showed a contiguous bundle through the tube, which was parallel to the growth axis in the control pollen tubes (**Figures 4A,A1**). However, under boron toxicity, the actin filaments were clearly twisted and condensed. The disrupted actin filament fragments accumulated into clusters with a very strong signal in the apical region (**Figures 4B,B1**, **Supplementary Figure S1b**).

### Effect of Boron Toxicity on Cellulose and Callose Deposition on the Pollen Tube Wall

**FIGURE 5:**
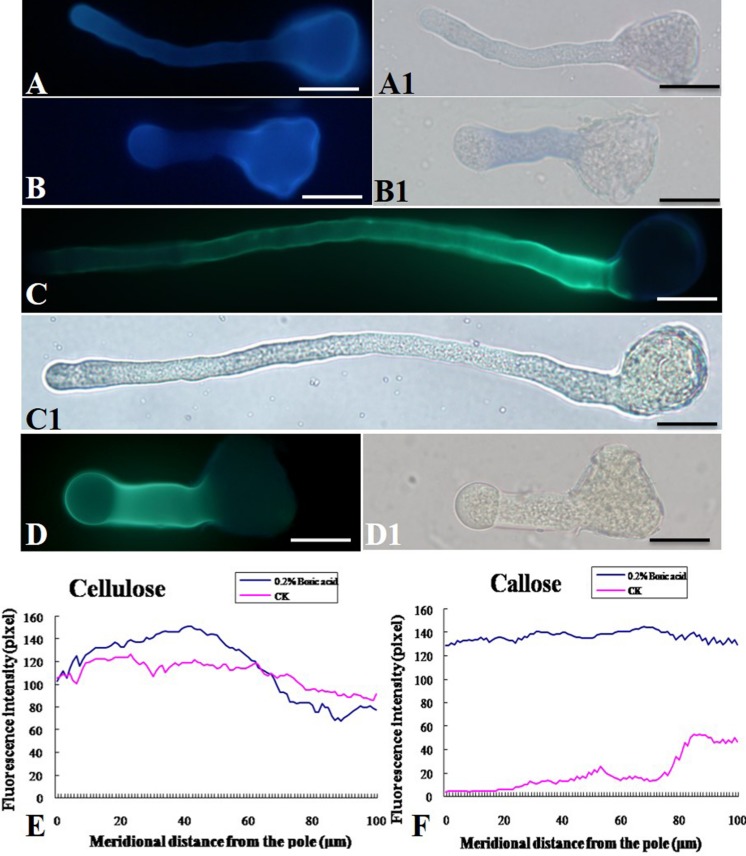
**Effect of boron toxicity on the distribution of cellulose and callosein the *Malus domestica* pollen tube. (A)** Cellulose distributed on the whole control pollen tube indicated by fluorescence of calcofluor. **(A1)** Corresponding bright field image of A. **(B)** Boron toxicity treated pollen tube showed more cellulose at the pollen tube tip indicated by fluorescence of calcofluor. **(B1)** Corresponding bright field image of B. **(C)** Callose was distributed along the entire length of the control pollen tube except the apex. **(C1)** Corresponding bright field image of C. **(D)** Strong fluorescence was detected along the entire pollen tube treated by boron toxicity, including the apex, indicating more callose accumulation at the apex under boron toxicity. **(D1)** Corresponding bright field image of D. **(E)** Quantitative analysis of the florescent signal of cellulose in the wall of control pollen tubes (CK, pink line) and tubes under boron toxicity (0.2% boric acid, blue line). **(F)** Quantitative analysis of the fluorescent signal of callose in the wall of control pollen tubes (CK, pink line) and tubes under boron toxicity (0.2% boric acid, blue line). Scale bar: 25 μm.

As shown in **Figure 5**, boron toxicity did not alter the distributive pattern and deposition of the cellulose in pollen tube wall (**Figures 5A,B,E**). Aniline blue staining showed that callose was present evenly along the tube except for the tip in control pollen tubes (**Figures 5C,C1**). On the contrary, strong fluorescence was observed in the pollen tube tip treated by boron toxicity, suggesting enhanced callose deposition at the tip in response to boron toxicity (**Figures 5D,D1,F**).

### Impact of Boron Toxicity on Pectin and AGP Deposition on Pollen Tube Wall

**FIGURE 6:**
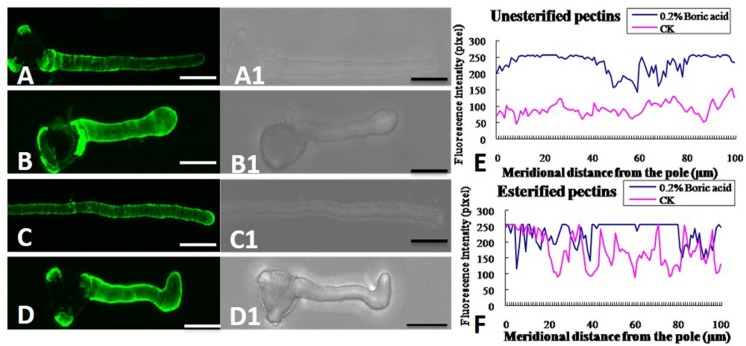
**Effect of boron toxicity on the distribution of acid and esterified pectins, respectively. (A)** Fluorescence by JIM5 labeling of control pollen tubes with much acid pectin at the basal part and less in the apex. **(A1)** Corresponding bright field image of a. **(B)** Fluorescence indicated more acid pectins in the apex of the boron toxicity-treated pollen tube. **(B1)** Corresponding bright field image of b. **(C)** Fluorescence from JIM7 labeling of control pollen tube with much esterified pectin in the apex. **(C1)** Corresponding bright field image of c. **(D)** Fluorescence was observed evenly along the tube after antibody JIM7 labeling of pollen tubes in the presence of boron toxicity. **(D1)** Corresponding bright field image of d. **(E)** Quantitative analysis of the fluorescence signal of acid pectins in the wall of control pollen tubes (CK, pink line) and tubes under boron toxicity (0.2% boric acid, blue line). **(F)** Quantitative analysis of the fluorescence signal of esterified pectins in the wall of the control pollen tubes (CK, pink line) and tubes under boron toxicity (0.2% boric acid, blue line). Scale bar: 25 μm.

In the control pollen tubes, the distribution of JIM5-labeled (de-esterified or acid) pectin was relatively uniform, with much at the basal part near the grain and less at the tip (**Figures 6A,A1**), whereas the localization of JIM7-binding (esterified) pectin was relatively uniform, with stronger fluorescence at the apex of the growing tubes (**Figures 6C,C1,E**). Both types of pectin showed a polar distribution. By contrast, more de-esterified pectin was detected along the entire tube (**Figures 6B,B1**) and more esterified pectin was detected on the entire pollen tube under boron toxicity (**Figures 6D,D1,F**). That is, no obvious polar distribution of pectin was observed in these pollen tubes.

**FIGURE 7:**
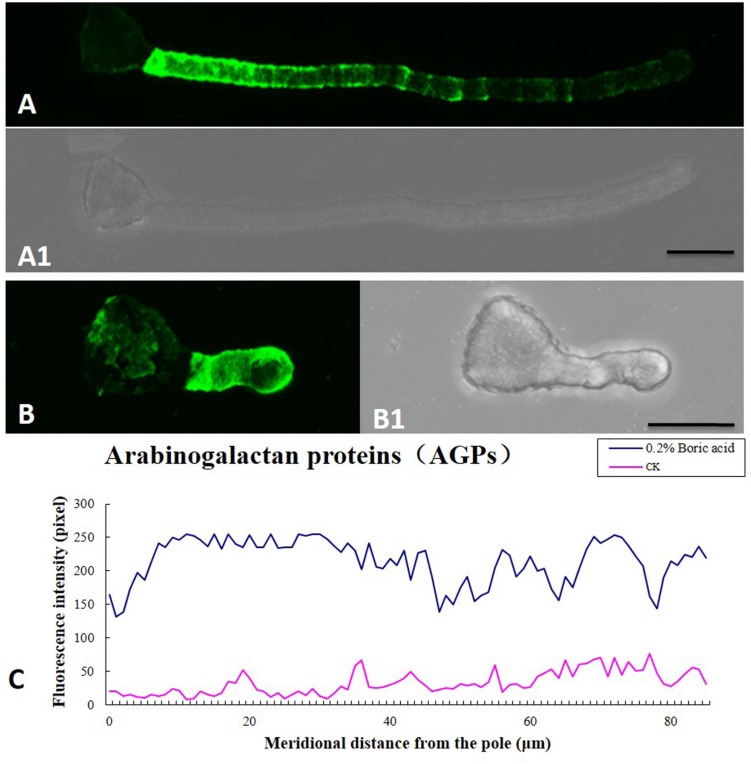
**Influence of boron toxicity on the distribution of arabinogalactan proteins (AGPs) in *Malus domestica* pollen tubes. (A)** Fluorescence after antibody LM2 labeling of pollen tubes cultured in normal medium, indicating more AGPs in the basal part and decreasing levels from the base to the pollen tip. **(A1)** Corresponding bright field image of **(A)**. **(B)** Fluorescence was observed throughout the entire tube; note the irregular distribution. **(B1)** Corresponding bright field image of **(B)**. **(C)** Quantitative analysis of fluorescence signal of AGPs in the wall of control pollen tubes (CK, pink line) and boron toxicity-treated tubes (0.2% boric acid, blue line). Scale bar: 25 μm.

Boron toxicity clearly disrupted the distribution pattern of AGPs. The typical characteristic AGP distribution on the pollen tube wall is a periodic ring-like pattern with a stronger signal in the basal part and weaker signals at the apex (**Figures 7A,A1**). However, under boron toxicity, the typical pattern disappeared. Instead, continued but irregular deposition was observed (**Figures 7B,B1**). Quantitative analysis of the fluorescence signal of AGPs in the wall indicated that boron toxicity induced more AGP accumulation in the pole of the tube (**Figure 7C**).

### FTIR Spectroscopy Analysis of Pollen Tube Wall Components

**FIGURE 8:**
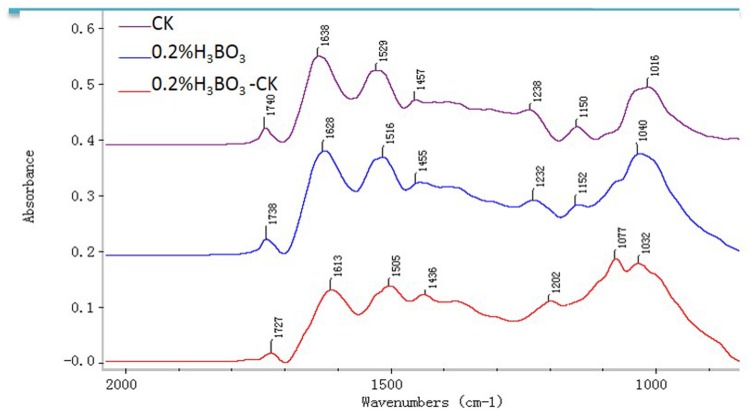
**Fourier-transform infrared analysis spectra from the tip regions of *Malus domestica* pollen tubes (control pollen tube: CK; pink), pollen tubes treated with 0.2% boric acid medium (blue, 0.2% H_3_BO_3_) and the FTIR differential spectrum (red) generated by digital subtraction of the pink spectra (CK) from the blue spectra (0.2% H_3_BO_3_)**.

Representative FTIR spectra gained from the tip domain of control and 0.2% boric acid-treated pollen tubes are shown in **Figure 8**. For the control pollen, saturated esters absorbed at 1740 cm^–1^, amide stretching bands of proteins absorbed at 1638 and 1529 cm^–1^, carboxylic acid groups absorbed at 1457 cm^–1^, and carbohydrates absorbed between 1200 and 900 cm^–1^. In the presence of 0.2% boric acid, the ester peak at 1738 cm^–1^ was increased, free acid stretched at 1455 cm^–1^, and the amide stretching bands of proteins absorbing at 1628 and 1515 cm^–1^ were increased, indicating that pollen tubes under boron toxicity showed increased esterified pectins, acid pectins and AGPs compared with the values in normal pollen tubes.

## Discussion

Boron toxicity has been report to affect various developmental processes in plants (Nable et al., 1990, 1997; Reid, 2007; Guo et al., 2014). Baluška et al. (2003) reported that interactions between pectins, boron, and the cytoskeleton were important for the assembly of the cell wall-cytoskeleton continuum as well as for its maintenance via signal-mediated processes. So the changes in boron concentrations may cause to a mechanical cascade of signals extending into the cytoplasm via the cell wall-plasma membrane-cytoskeleton continuum, with the possible involvement of AGPs (Camacho-Cristóbal et al., 2008). The hypothesis is sustained by the researches that boron deficiency resulted in an varied polymerization pattern of cytoskeletal proteins (Yu et al., 2001, 2003) and in inhibition of the endocytic pathway for the internalization of B-cross-linked RG-II pectins (Yu et al., 2002). Our present work provides novel evidence for this proposal.

### Boron Toxicity Induced a Decrease of [Ca^2+^]c and Disappearance of the [Ca^2+^]c Gradient

It has been appreciated that Ca^2+^ plays a key role in determining the structure and function of the cell wall (Hepler, 2005). The apical wall of normal pollen tube consists almost entirely of pectin, with cellulose and callose being located behind the apex (Ferguson et al., 1998). Ca^2+^ affected the mechanical properties of the cell wall through cross-linking de-esterified HG of pectin (Peaucelle et al., 2012). As a consequence, when the [Ca^2+^] is lowered sufficiently the pollen tube wall loses its structural integrity and therefore bursts, whereas when [Ca^2+^] is high, the pectin chains will be cross-linked and aggregated, and the wall maximally rigidified (Hepler and Winship, 2010). Picton and Steer (1983) stated that the permissive [Ca^2+^] for pollen tubes extends between 10 μM to10 mM. Because 20–30% of the newly deposited pectin will be de-esterified, there will be always an immediate need for Ca^2+^ by the growing pollen tube (Hepler and Winship, 2010). Ca^2+^ has been reported to be involved in the signal transduction pathway of boron deficiency (González-Fontes et al., 2014), which was supported by the reports that boron deficiency increased the levels of cytosolic Ca^2+^ in tobacco BY-2 cells (Koshiba et al., 2010) and *Arabidopsis thaliana* roots (Quiles-Pando et al., 2013).

In the present study, we revealed that boron toxicity induced a decrease in [Ca^2+^]c concentration and a disappearance of the [Ca^2+^]c gradient, suggesting the sensitive and critical role of Ca^2+^ in boron signaling in the proposed mechanical cascade of signals which extended from cell wall to the cytoplasm via the cell wall-plasma membrane-cytoskeleton continuum (Camacho-Cristóbal et al., 2008). Calcium is likely a major element in transmitting boron signals and modulating cytoplasmic activities. We speculate that excessive boron first binds to pectin in the wall of the pollen tube and provides many more binding sites for calcium, which results in a [Ca^2+^]c decrease and the disappearance of the calcium gradient in the tip of the pollen tube, resulting in irregular pollen tube growth. Although more evidence is needed to support this proposal, our results indicated that calcium might be involved in the responses to boron toxicity.

### Boron Toxicity Disturbed the Actin Filaments

Boron deficiency induced alteration of cytoskeleton biosynthesis (Yu et al., 2001, 2002), suggesting that a linkage between boron and cytoskeleton may exist. Previous evidence indicates that actin filaments play an essential role in the transport of secretory vesicles and pollen tube growth (Cárdenas et al., 2008; Fu, 2015; Qu et al., 2015). In normal pollen tubes, actin filaments are reported to be arrayed in bundles and extend the subapical region (Shi and Yang, 2010). It was reported that there exists crosstalk between calcium signaling and cytoskeleton in the pollen tube, while Ca^2+^ is a central factor controlling the transition from G-actin in the tube apex to the F-actin cables in the shank (Shi and Yang, 2010). These results suggest that Ca^2+^ is critical for the actin organization in pollen tubes.

In the present research, we have revealed that actin organization is sensitive to boron toxicity. Both the specialized structure and distribution were clearly disturbed in pollen tubes under boron toxicity. This abnormal appearance of actin organization was coupled with decreased [Ca^2+^]c and the disappearance of the [Ca^2+^]c gradient in pollen tubes under boron toxicity. Based on the previous findings noted above, it is reasonable to speculate that actin organization abnormality might result from boron toxicity-induced low [Ca^2+^]c. It is likely that boron toxicity signaling is mediated by calcium dynamics to achieve the cytoplasmic response.

### Boron Toxicity Altered the Deposition of Pollen Tube Wall Components

Boron toxicity affects the morphology of pollen tube, thus we want to know whether the tube wall composition was affected by boron toxicity. Results showed that cellulose was present throughout the pollen tube wall under normal conditions and under boron toxicity. Boron toxicity showed no obvious effect on the cellulose deposition of the pollen tube. Callose can be synthesized in the normal pollen tube walls (Qin et al., 2012). In addition, callose is distributed at the tips of abnormal pollen tubes (Hao et al., 2013). Our results showed that boron toxicity altered the deposition pattern of the callose in the pollen tube walls of *Malus domestica*. In the control pollen tube, callose was detected along the entire pollen tube except for the tip, but in the presence of boron toxicity, callose was distributed along the entire tube including the tip.

Beside cellulose and callose, pectin is an important composition of the pollen tube wall. Pectin is secreted mainly as methoxy-esters, and later de-esterified t by the enzyme pectin methyl esterase (PME) (Bosch and Hepler, 2005; Peaucelle et al., 2012). Plant cell wall also contains essential minerals including calcium and boron, which are necessary for formation of networks of pectic polysaccharides in cell walls. The extent and strength of Ca^2+^ cross-linking depend on the acidic residue of the de-esterified pectins (Hepler and Winship, 2010). Research by Fang et al. (2008) illustrated that pectin associates with carboxyl moieties which participate in binding with free Ca^2+^ to form plastic gels. Ngouémazong et al. (2012) reported that at high [Ca^2+^], with a low degree of methoxylation, pectins reach maximum strength. According to the previous studies, pollen tube growth is speculated to depend on a balance between the number of available acidic residues and the [Ca^2+^]. That is too few acidic groups or too little Ca^2+^ will lead to the pollen tube burst, but too many acidic groups and/or too much Ca^2+^ will result in overly cross-linked wall and therefore pollen tube can’t extend (Hepler and Winship, 2010). Our immunolabeling results showed that in the boron toxicity-treated pollen tube, there was more acidic pectin, which could create more binding sites for calcium and thus result in less calcium ion in the cytoplast. Therefore, the ratio of de-esterified pectin to esterified pectin plays a critical role in the adjustment of the cytoplasmic calcium level and in the transmission of boron toxicity-induced effects on pollen tube growth.

Arabinogalactan proteins are proteins which exist in plant and distribute through different developmental stages (Pereira et al., 2014). AGPs can interact with pectins or other cell wall-localized proteins (Baldwin et al., 1993; Showalter, 2001). Recent researches have enhanced our understanding of AGP’s role in plant (Lamport and Várnai, 2013; Lamport et al., 2014). AGPs may play an essential role in the boron deficiency signal transduction by binding Ca^2+^ (González-Fontes et al., 2014). In the present study, AGPs were deposited by LM2, indicating that boron toxicity caused AGPs to accumulate throughout the pollen tube except the basal part near the grain, instead of the characteristic periodic ring-like deposits with less signal at the tip. Boron toxicity changed the distribution pattern and quantity of AGPs, which may interlink with calcium and actin alteration. More AGPs might bind to more calcium and result in low [Ca^2+^]c. These findings indicated that boron toxicity induced reconstruct of tip cell wall components, resulting in cell wall rigidity/extensibility changes and subsequent slowing and cessation of growth.

It can be expected that boron toxicity alters the cell wall structure, and a rapid change in the mechanical strength of the cell wall occurs. This effect triggers a mechanical cascade of signals through the cell wall-plasma membrane-cytoskeleton continuum, in which AGPs most likely take an active part (Goldbach and Wimmer, 2007). This expectation agrees with the structural modification of the cell wall pectin and cytoskeleton under boron toxicity in this research.

Boron toxicity is an important agricultural problem that limits crop productivity, however, under boron stress condition, how much boron could accumulated in style and directly regulate pollen tube growth remains unknown. Therefore, *in vitro* test the influence of the high boron on pollen tube growth will provide useful clue to understand possible response of pollen tube to the stress.

In the low rainfall and on highly alkaline and saline soil where the rate of boron is over 2.0 mg/L, there is boron pollution and consequently decreases in production and defect in the products can be seen (Ozturk et al., 2010). When boron is present at high concentrations in the soil or ground water, plant growth, and reproduction can be affected by boron toxicity (Roessner et al., 2006). So boron toxicity has been recognized as an important problem limiting crop production. Following long-term exposure to high B concentrations, overall vegetative plant growth is retarded and this leads to either a reduction in or a complete lack of seed set (Roessner et al., 2006).

Application of boron fertilizer in soil and boron foliar application appear worthwhile in the field. Both of the methods increased the boron concentration in various parts of the plant (Asad et al., 2003). There are no data on boron concentration in style and how much boron pollen tube could absorb from style so far. However, based on the previous study mentioned above, the concentration of boron in style increases if the plant grows under high boron. Whether the boron concentrations used in the present study is similar to the *in vivo* values need further research.

Our *in vitro* test illustrated that 0.2% boron inhibited *Malus domestica* pollen germination and arrested pollen tube growth. Based on this, if boron foliar application or boron in the soil at high concentrations leads to similar boron concentration in style, it will be harmful to pollen tube growth.

In brief, our investigation of the effects of boron toxicity on *Malus domestica* pollen tubes provides an extensive understanding of the role of boron in the polarized tip growth of pollen tubes. We found that boron toxicity decreases [Ca^2+^]c, inducing the disappearance of the [Ca^2+^]c gradient and altering actin filament organization. The distorted actin filament may disturb transport of the wall precursor to the pollen tube wall, resulting in defects in cell wall construction and variations in pollen tube tip growth. This research provides new insights into the boron function in pollen tube growth and valuable evidence for the previous proposal that boron may lead to a mechanical cascade of signals from the wall to the cytoplasm through the cell wall-plasma membrane-cytoskeleton continuum.

## Author Contributions

The authors have made the following declarations regarding their contributions: Conceived and designed the experiments: KF. Performed the experiments: WZ, KF. Analyzed the data: KF, WZ, YX, QZ, LY, and LQ. Contributed to the writing of the manuscript: KF, WZ, YX, QC, and LQ.

## Conflict of Interest Statement

The authors declare that the research was conducted in the absence of any commercial or financial relationships that could be construed as a potential conflict of interest.

## Supplementary Material

FIGURE S1 **(a)** Weak fluorescence was detected at the apex of the pollen tube under boron toxicity for 1.5 h, no Ca gradient was visible. **(b)** The actin deposition varied upon treatment with high boron for 1.5 h.
