# Supplementary figures and images for: Boron Toxicity Causes Multiple Effects on Malus domestica Pollen Tube Growth
Source: Front Plant Sci. 2016 Feb 26;7:208. doi: 10.3389/fpls.2016.00208 (PMC4768074; doi:10.3389/fpls.2016.00208)

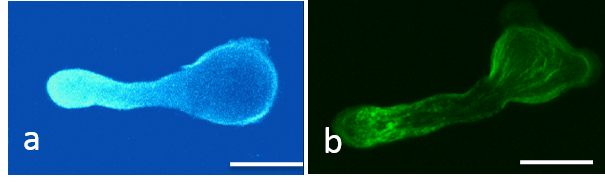

Supplement: FIGURE S1 — (a) Weak fluorescence was detected at the apex of the pollen tube under boron toxicity for 1.5 h, no Ca gradient was visible. (b) The actin deposition varied upon treatment with high boron for 1.5 h. [file Image_1.TIF]
